# Diagnosing Vestibular Hypofunction in Children with Sensorineural Hearing Loss: Using the Video Head Impulse Test or the Caloric Test First Not the Cervical Vestibular Evoked Myogenic Potential

**DOI:** 10.3390/jcm14082721

**Published:** 2025-04-15

**Authors:** Max Gerdsen, Britt Gerrianne Schuurman, An Boudewyns, Raymond van de Berg, Josine Christine Colette Widdershoven

**Affiliations:** 1 Division of Vestibular Disorders, Department of Otorhinolaryngology and Head and Neck Surgery, Maastricht University Medical Center+, 6229 ET Maastricht, The Netherlandsraymond.vande.berg@mumc.nl (R.v.d.B.);; 2 Department of Otorhinolaryngology and Head and Neck Surgery, Antwerp University Hospital, 2650 Edegem, Belgium; 3 Faculty of Medicine and Translational Neurosciences, Campus Drie Eiken, University of Antwerp, 2610 Wilrijk, Belgium

**Keywords:** children, sensorineural hearing loss, vestibular, video head impulse test, caloric test, cervical vestibular evoked myogenic potential

## Abstract

**Background/Objectives:** Children with sensorineural hearing loss (SNHL) can develop, or have concurrent vestibular hypofunction (VH). Assessing the vestibular function is challenging in the pediatric population. The objective of the current study was to identify the most effective test battery for objectively diagnosing and screening VH in children with SNHL. **Methods**: A two-center retrospective chart review included 71 children aged six months to 18 years old with unilateral or bilateral SNHL. Testing consisted of the video head impulse test (VHIT), the caloric test and cervical vestibular evoked myogenic potential (cVEMP). Pairwise agreement between tests was calculated by the proportion of overall agreement and unweighted Cohen’s kappa. **Results**: Vestibular hypofunction was diagnosed less often by cVEMP compared to VHIT or the caloric test. The overall disagreement observed between VHIT and cVEMP and the caloric test and cVEMP was explained by a higher proportion of ears diagnosed with VH by VHIT (18 versus four) or the caloric test (14 versus 0). Several cases with normal cVEMP responses had abnormal test results for VHIT (18 of 71 ears) or the caloric test (14 of 32 ears). VHIT and the caloric test showed a moderate inter-test agreement (Kappa 0.591; *p* = 0.018). **Conclusions**: VHIT and the caloric test had a higher likelihood of diagnosing VH, as opposed to cVEMP. It would therefore be advised to use VHIT or the caloric test as the first-line vestibular test for children with SNHL to screen for VH. The clinical value of cVEMP seems low in children with SNHL.

## 1. Introduction

Children with sensorineural hearing loss (SNHL) can develop or have concurrent vestibular hypofunction (VH). The reported prevalence is between 20% and 70% [1,2,3,4,5]. This prevalence can be explained as both SNHL and VH usually have a common etiology due to their embryological and anatomical relation [1]. Children affected by VH are often unrecognized due to their limited communicative abilities and atypical presentation of vestibular symptoms. Examples of this presentation are delayed motor development milestones, frequent falls, and impaired writing, reading or learning skills, leading to delayed cognitive development [2,3,4,5]. The early detection of VH enables adequate counseling of parents and early vestibular rehabilitation. Moreover, objective information on vestibular function may contribute to decision-making for cochlear implantation or vestibular implantation in the future [6,7,8,9].

The video head impulse test (VHIT), caloric test and cervical vestibular evoked myogenic potential (cVEMP) are commonly used tests for peripheral vestibular function [10]. Each of these tests addresses a different function or component of the vestibular organ, such as the various frequencies of the semicircular canals or otolith function [7,11,12,13]. VHIT is a quickly performed test and is able to assess all semicircular canals, whereas the caloric test assesses specifically the horizontal semicircular canal and superior vestibular nerve. However, VHIT shows high interobserver variability, and the most commonly available equipment is adapted to adults. The aforementioned tests are commonly performed in, and adapted to, adults. Performing these tests is more challenging in the pediatric population. The majority of vestibular tests are hard to reproduce and not well tolerated. This is mainly attributed to the tests being time-consuming and, in some cases, the aggravation of vestibular symptoms. Small adjustments are made to reduce these challenges. Nevertheless, only a limited number of centers have enough experience to perform reliable pediatric vestibular testing.

Previous studies assessed the quality of vestibular testing in children. In a systematic review by Verbecque et al., the cVEMP, caloric test and VHIT were assessed for sensitivity and specificity in determining vestibular function [10]. It was found that the diagnostic accuracy of the various tests differed substantially between studies. Moreover, no consensus exists on an appropriate pediatric test battery; whereas one study has claimed that VHIT and cVEMP should be performed [13], another study has claimed that a combination of the rotatory chair test and the caloric test is most optimal [14]. The VHIT is effective and often well tolerated by children. However, the main disadvantage of VHIT is the necessary visual acuity of the child to focus on the presented target. This limitation is not present for the cVEMP, which can be performed at any age. The caloric test is often regarded as the gold standard for vestibular testing. However, it can be frightening for children, as vision is denied during the examination, and the irrigations can cause pain, dizziness and nausea [11,15,16]. Moreover, the caloric test is more time-consuming compared to other tests [12]. Altogether, there are numerous tests that investigate different components of the vestibular organ, and each test has advantages and disadvantages in its applicability to the pediatric population [7,13,14,17]. A common consensus is that these tests address different physiological responses of the vestibular organ [5]. Therefore, each test could be complementary or essential in addressing potential VH [18].

A recent study conducted by our group explored the diagnostic accuracy of combining multiple vestibular tests in children with SNHL and compared the results to an expert clinical evaluation. It was concluded that a test battery should consist of at least the VHIT or the caloric test. It could be complemented with cVEMP if the primary test outcome was normal [19]. While this approach demonstrated promise, it is important to acknowledge that there is no established gold standard for diagnosing VH in children. This lack of a gold standard consequently shows the limitations of the assessment process, as the vestibular diagnosis is subjected to clinical interpretation by a pediatric vestibular specialist. There is a scarcity of centers with clinical expertise in pediatric vestibular disorders. Therefore, there is a need to develop a diagnostic algorithm designed specifically for screening of VH in this population. This algorithm should minimize subjective interpretation, allowing broader application in clinical settings without the required resources. For it to be potentially routine clinical practice, the algorithm must be reliable, time-efficient and minimally burdensome for the child.

The primary objective of the current study was to identify the most effective test battery for objectively diagnosing and screening for VH in children with SNHL. Redundancy in testing should be avoided, as performing multiple tests on the same patient may be unnecessary if their outcomes are positively correlated. Therefore, the inter-test agreement between VHIT, the caloric test and cVEMP was assessed. In addition to evaluating agreement, tests with a higher likelihood of detecting VH would be prioritized as the primary diagnostic tool. By combining these findings, an objective diagnostic algorithm that minimizes test redundancy and ensures accurate detection of VH in pediatric populations is proposed.

## 2. Materials and Methods

### 2.1. Study Population

This retrospective chart review included patients between 2019 and 2022 from the outpatient clinic of the Ear, Nose and Throat Department of Maastricht University Medical Centre and Antwerp University Hospital (approved by the local Ethics Committee; 2019-1215 and 20/14/165, respectively). Children with congenital or acquired SNHL were included. Exclusion criteria were children with test results after cochlear implantation and traumatic SNHL. Patients were selected from the same cohort as the previous study [19]. Seventy-one children were included between the ages of six months and 18 years old with unilateral or bilateral SNHL or mixed hearing loss. Hearing loss was defined by a pure-tone average of 30 decibels or more at the frequencies of 500, 1000, 2000 and 4000 hertz in the unaided situation (hearing aid). For children under the age of two and a half, hearing loss was assessed using otoacoustic emissions or visual reinforcement audiometry. For children over the age of two, play audiometry was conducted whenever possible. Subsequently, Brainstem Evoked Response Audiometry or otoacoustic emissions were performed to objectively determine the degree of hearing loss.

### 2.2. Vestibular Testing

Physical examination of the ear, nose, and throat was conducted before vestibular testing. Motor function was assessed by evaluating locomotion, the one-leg standing test with eyes open and closed, and the Romberg test if the patient was able to perform the tests. Subsequently, the oculomotor function was examined by assessing a range of eye movements, smooth pursuit, optokinetic nystagmus, spontaneous nystagmus, saccades and vergence [20]. Subjects with oculomotor function issues or signs of central nervous system pathology were excluded from further testing. The available peripheral vestibular tests in both centers were VHIT, the caloric test and cVEMP. Test results were reported for each ear per subject. Rotatory chair testing (torsion swing test) was excluded as this test does not assess the vestibular function of a single ear specifically, as opposed to the included vestibular tests [21]. The tests were carried out in a randomized sequence to reduce the influence of fatigue or attention loss during testing. Trained examiners, one in each center, performed the tests. The examiners determined whether the test was performed correctly and whether the measurements were reliable. The test results were interpreted by the same pediatric otolaryngologist in both centers (J.W). At least two tests were performed successfully on both ears per subject.

The Synapsys system (Ulmer, Marseille, France) for VHIT was used in both centers. This system registers the eye and head movements with a high-speed infrared camera. In some children above the age of 12, a different device was used (ICS impulse system, Otometrics-Natus, Taastrup, Denmark). This system uses goggles with a built-in high-speed infrared video camera, which records eye movements. In addition to the camera, the goggles also contain a gyroscope, which records head movement and velocity [11]. The applied testing protocol was described by Wiener-Vacher and Wiener [22]. A trained examiner stood behind the subject and rotated the head 30 degrees in the horizontal plane with a sudden and quick movement (head impulse) [13,23]. The vertical planes were not assessed because these were not conducted in younger children due to discomfort and poor tolerance. The head impulses were successful if the peak head velocity ranged between 150 and 400°/s. In children younger than four years, at least five well-executed impulses had to be registered per side [24]. In children older than four years, this amount was 10 impulses on both sides. The cut-off point for the gain of the vestibular-ocular reflex was set at 0.7. This was based on the available literature and after validating the test protocol at Maastricht University Medical Center [25].

Caloric testing was performed with two systems, namely Variotherm 3 (Atmos, Lenzkirch, Germany) at Maastricht University Medical Center and KALORIstar Arctic 1 (Biomed, Jena, Germany) at Antwerp University Hospital. The testing protocol was described by Janky and Rodriguez [11]. During this test, the subject lies in the supine position on a bed with the head tilted at 30 degrees [11,21]. At Maastricht University Medical Center, the examiner irrigated at least 300 mL of water at 34 and 40 °C for 30 s in each external ear canal. Testing in Antwerp University Hospital was performed with an airflow of 5–8 L/min at 25 and 44 °C for 60 s. During and after irrigation, eye movements were recorded by electronystagmography. The caloric test was only performed on subjects aged four years or older, due to the burden of the test on children [19]. No normative data for the caloric test were available. However, since it was hypothesized that the caloric response maturates before the age of four [11], caloric responses were compared to normative data of healthy adults. At the Maastricht University Medical Center, a normal caloric response was defined as a velocity of at least 12°/s during warm irrigation and 5°/s during cold irrigation. For Antwerp University Hospital, the corresponding thresholds for a normal response were 10°/s for warm irrigation and 5°/s for cold irrigation. Asymmetry between vestibular organs was calculated with the Jongkees’ formula [26].

Eye movements during oculomotor tests and caloric tests were recorded with electronystagmography. Adhesive electrodes (White Sensor ECG electrodes, Ambu, Copenhagen, Denmark) were fixed bitemporally, on the interior canthi and on the infra- and supraorbital margin of both eyes, to monitor eye movements [12]. Subjects below the age of two years had a similar setup around one eye. Electronystagmography for eye movements was recorded in both centers with KingsLab 1.8.1 (Maastricht University, Maastricht, The Netherlands) at Maastricht University Medical Center and Nystagliner (version 4.03, Toennies, Germany, or BalanceLab, Maastricht Instruments BV, Maastricht, The Netherlands) at Antwerp University Hospital.

CVEMP followed established procedures with the Neuro-Audio DI200300 EMG System (Version 2010, Neurosoft, Ivanovo, Russia) [12]. Self-adhesive electrodes (White Sensor ECG electrodes, Ambu, Copenhagen, Denmark) were fixed on four locations, namely the sternocleidomastoid muscles, sternum (reference electrode), and forehead (ground electrode). A 500 Hz tone burst of 59 dBnHL (129 dB SPL) was delivered by bone conduction on the mastoid with a frequency of 5 Hz for 100 clicks. Patients were positioned supine with their head inclined at a 30-degree angle from the horizontal plane and turned contralaterally from the stimulated side for contraction of the ipsilateral sternocleidomastoid muscle. Visual stimulation, such as showing videos on a tablet or presenting a favorite toy, was used to encourage patient cooperation. Each ear underwent testing at least twice to ensure reproducibility. The interpretation of cVEMP responses as ‘normal’ or ‘abnormal’ followed the criteria outlined by Martens et al. [27]. In summary, responses were considered normal if they exhibited at least two reproducible biphasic P1-N1 waveforms. The waveforms’ amplitude had to meet the cut-off values outlined in the referenced protocol to be considered a normal response. Additionally, a test result was deemed abnormal if latencies surpassed the threshold, defined as exceeding one standard deviation above the mean (P13 > 18.03 ms, N23 > 24.58 ms).

### 2.3. Data Analysis

Descriptive statistics were used to calculate participant characteristics. The Shapiro–Wilk test was used to assess the normality of the distribution of age. This concluded a non-normal distribution and was therefore reported as the median and interquartile ranges. VH was diagnosed in an ear if one of the vestibular tests showed an abnormal response. The inter-test agreement between the two tests was determined for each ear, ensuring that the group characteristics were similar across test results. This approach helps control for the potential influence of these variables on the results. To determine pairwise agreement between the different vestibular tests, the proportion of overall agreement was calculated. In addition, unweighted Cohen’s kappa was calculated to correct for a chance of agreement. The strength of agreement was labeled as ‘poor’ (kappa < 0.00), ‘slight’ (kappa 0.00–0.20), ‘fair’ (kappa 0.21–0.40), ‘moderate’ (kappa 0.41–0.60), ‘substantial’ (0.61–0.80), or ‘almost perfect’ (0.81–1.00). The prevalence index and bias index were presented separately for further interpretation of the chance of agreement [28]. The prevalence index presented the difference between the proportions of cases classified as ‘normal’ or ‘abnormal’ vestibular function by both tests. The bias index showed the extent to which the tests disagreed on the proportions of cases showing normal or abnormal vestibular function. All statistical analyses were performed with SPSS statistics version 27 (IBM Corp, Armonk, NY, USA).

## 3. Results

### 3.1. Participants

Table 1 depicts the baseline characteristics of the cohort. Seventy-one children were included in the study: 31 males and 40 females. The median age of the participants was 67 months (interquartile range 31–96.5 months). The majority of children had bilateral SNHL (70.4%). Forty-one (57.7%) subjects showed an abnormal vestibular response (indicating VH) in at least one of the three vestibular tests for at least one ear. Not all subjects with unilateral hearing loss presented with VH on the affected side.

### 3.2. Overall Agreement

Table 2 depicts the overall agreement between different vestibular tests. The VHIT and caloric test showed the highest overall agreement (87.5%), whereas the caloric test and cVEMP were the lowest (66.7%). For all combinations, the most common agreement was observed for determining normal vestibular function. Agreement in diagnosing VH was the lowest for VHIT and cVEMP (1.3%). Table 3a–c illustrates the distribution of diagnosing normal vestibular function or hypofunction between the different combinations of vestibular tests per ear. Overall, cVEMP diagnosed VH less often compared to VHIT or the caloric test. CVEMP diagnosed VH in five and VHIT in 19 of 76 ears that underwent both tests (Table 3b). In the case of cVEMP and caloric tests, cVEMP identified 10 cases of VH and the caloric test 24 in 42 ears (Table 3c).

The overall disagreement observed between VHIT and cVEMP (28.9%; Table 2) and the caloric test with cVEMP (33.3%; Table 2) is explained by a higher proportion of ears diagnosed with VH by VHIT (18 versus 4) or the caloric test (14 versus 0) as opposed to cVEMP (Table 3b,c). Furthermore, if VHIT or the caloric test showed a normal test result, the chance of an abnormal test result for cVEMP was low (4 of 57 ears and 0 of 18 ears, respectively). However, in the case of cVEMP presenting a normal test result, there was a higher chance of concluding an abnormal test result for the VHIT or the caloric test (18 of 71 ears and 14 of 32 ears, respectively) (Table 3b,c). This suggests that VHIT and the caloric test may be more sensitive to certain vestibular deficits than cVEMP.

### 3.3. Inter-Test Agreement

The inter-test agreement is presented in Table 4. Both combinations of the caloric test with VHIT and cVEMP showed a significant inter-test agreement (kappa 0.591; *p* = 0.018 and kappa 0.380; *p* = 0.002, respectively). VHIT and the caloric test showed moderate agreement, supporting the notion that these tests provide relatively consistent results when diagnosing VH. The caloric test and cVEMP had a fair agreement, indicating a weaker inter-test consistency, which is likely due to cVEMP diagnosing VH less frequently (Table 3b,c). The VHIT and cVEMP demonstrated poor agreement (Kappa −0.023; *p* = 1.000). This finding suggests and reinforces the assumption that both tests assess different vestibular pathways and do not consistently yield similar diagnoses. The high prevalence index for VHIT and the caloric test (0.625) and for VHIT and cVEMP (0.684) suggests that the low agreement in diagnosing VH contributes significantly to the overall variability (Table 2). Additionally, the high bias index for cVEMP with both VHIT (0.184) and the caloric test (0.333) indicates that cVEMP underestimates the prevalence of VH compared to the other tests. These discrepancies indicate that VHIT and the caloric test may be more sensitive to detecting VH, while cVEMP may be more specific but less sensitive.

## 4. Discussion

The objective of this study was to propose an objective diagnostic algorithm for screening and detecting VH in the pediatric population with SNHL. Vestibular hypofunction was highly prevalent in the tested cohort (57.7%), consistent with the existing literature [5,17,29,30,31]. The combinations of the caloric test with VHIT or cVEMP showed a significant inter-test agreement, suggesting that combining these tests might be redundant in the vestibular assessment. Additionally, VHIT and the caloric test had a higher likelihood of diagnosing VH, as opposed to cVEMP, indicating that the primary test should be the caloric test or VHIT.

The overall disagreement between cVEMP with both VHIT and the caloric test could be explained by the lower likelihood of diagnosing VH by cVEMP. The chance of missing VH with a concomitant vestibular test was noticeably higher for cases with a normal test result for cVEMP. An explanation could be that detecting VH with a single-frequency cVEMP is potentially less sensitive compared to multi-frequency VEMP testing [32]. A second explanation could be that children with congenital or acquired vestibular pathology more often presented with hypofunction of the horizontal semicircular canals as opposed to the saccule [33]. For example, a proportion of the included children were diagnosed with congenital cytomegalovirus infection. The available literature showed that subjects with confirmed congenital cytomegalovirus infection presented more often with deterioration of the semicircular canal function compared to saccular function [34,35,36]. However, other studies presented opposite findings for children with VH overall. Two studies observed a higher chance of decreased otolith responses than semicircular canal responses. These studies compared cVEMP with the caloric test and rotatory chair test, consistently demonstrating a greater incidence of abnormal cVEMP responses compared to both [16,37]. To summarize, the overall low diagnosis of VH by cVEMP could be due to a lower sensitivity of single-frequency cVEMPs or a lower presence of saccular pathology in this cohort.

To our knowledge, this is the first study that assessed the inter-test agreement between these three vestibular tests in pediatric patients. Regarding the caloric test and cVEMP, no significant correlation was found in adult patients with acoustic neuroma or definite Ménière’s disease [38]. This finding does not correspond with the observed positive agreement in this study. Regarding VHIT and caloric tests, no correlation and low overall agreement were found in the literature [39,40,41,42,43]. This does also not align with the presented data in this article, as the inter-test agreement was significant and the overall agreement was moderate. Regarding VHIT and cVEMP, only a positive agreement was observed in the literature between VHIT of the posterior semicircular canal and cVEMP. This was not assessed in this study, as only data on VHIT of the lateral semicircular canal was included [44,45,46]. The discrepancy between cVEMP on the one hand and the caloric test and VHIT on the other hand could be explained by the difference in stimulation targets. CVEMPs measure the otolith function, while the caloric test and VHIT measure the semicircular canals. This assumption is strengthened by the stronger agreement between VHIT and the caloric test. Additionally, disease-specific factors can lead to variations in test outcomes. For example, VHIT and the caloric test exhibited discordant results in patients with Ménière’s disease but demonstrated an agreement in cases with confirmed vestibular neuritis [43]. Overall, different parts of the vestibular system could be affected differently by specific vestibular disorders, as stated above [47].

A possible explanation for the discrepancies in the observed outcomes between the literature and this study regarding inter-test agreements could be the differences in methodological setup. This study included pediatric patients based on SNHL, whereas the available literature selected patients based on vestibular symptoms or diagnoses. Therefore, the population of this study differed substantially from the literature, as VH was only present in 57.7% of the subjects, as opposed to 100% in the literature [38,39,40,41,42,44,45,46]. Nonetheless, the aim of this study was to assess the agreement in a population representing routine clinical practice. The included population was therefore considered representative of the objective of this study.

### 4.1. Limitations

In this study, several limitations were identified. Firstly, due to the retrospective nature of this study, not all vestibular tests were performed on each subject. The main reason was the child not being able to complete a test or multiple tests due to attention span or tiredness. The caloric test was not performed on children under the age of four. Furthermore, the testing protocol differed between both centers, as water irrigation was used in one center and air in the other, affecting the generalizability of the data. Due to the incomplete data of all vestibular tests for each subject, results might be affected by selection bias. The cohort was too small to assess this potential issue adequately. Secondly, it should be noted that not all commonly available vestibular tests were assessed in this study. VHIT could be complemented with tests of the vertical planes. This was, however, not performed in this cohort. The primary reason is the discomfort experienced by younger subjects during vertical plane testing. Therefore, the VHIT data in this study does not provide comprehensive information for all semicircular canals. Furthermore, the rotatory chair test was not included. This vestibular test addresses the low- to middle-frequency domains of the lateral semicircular canal [21]. Two modalities are commonly performed in this test, namely the torsion swing test and the velocity step test. Whereas the outcome measurements of the torsion swing test provide information on both lateral semicircular canals, the velocity step test mainly tests one semicircular canal. Therefore, the velocity step test produces information that addresses unilateral VH more specifically. The torsion swing test was mainly performed on the included subjects in this study and was therefore excluded. Thirdly, this study lacked the ability to evaluate potential false positives or false negatives due to the absence of established gold standards. The normative values and cut-off points for cVEMP and VHIT were derived from mean values with associated confidence intervals, inherently allowing for some margin of error in the test outcomes within the healthy population. Consequently, these limitations introduced a degree of uncertainty regarding the accuracy of the presented values for diagnosing VH in the studied population. These uncertainties could be mitigated by incorporating a control group. This would identify potential false positive and false negative rates. This knowledge would subsequently enhance the interpretation of the observed agreements between the tests. Overall, these limitations highlight the challenges of retrospective analysis and emphasize the existing difficulties in vestibular testing for children.

### 4.2. Clinical Implications

This study suggests a test battery that preferably includes at least the caloric test or VHIT to assess and screen for VH in children with SNHL. VHIT is preferred as a primary screening method for VH in children with SNHL. This consideration takes into account that VHIT is less time-consuming and more child-friendly compared to the caloric test. However, an important limitation of VHIT is the minimum age required of children that have sufficient visual acuity, a restriction not present in cVEMP testing. An alternative screening approach would be to perform cVEMP if the child is below the minimum required age, followed by VHIT at a later stage if cVEMP did not detect VH. However, it should be noted that the clinical value of cVEMP is disputable in children with SNHL: (1) cVEMP is inferior to VHIT and the caloric test to detect VH; (2) the added value of cVEMP is low if VHIT and/or the caloric test are already performed. Furthermore, the assumption that cVEMP exclusively assesses the function of the saccule or inferior vestibular nerve has recently been debated [48]. Therefore, its added value alongside VHIT or the caloric test remains uncertain. Therefore, a test battery should preferably include at least the VHIT or the caloric test.

## 5. Conclusions

In children with SNHL, it is recommended to use VHIT or the caloric test first to screen for vestibular hypofunction. The clinical value of cVEMP seems low in this population. Future studies should expand the test battery by incorporating the rotatory chair test, specifically the velocity step, or VHIT in vertical planes. These additions would provide more comprehensive advice on the most optimal test battery for screening VH in children with SNHL.

## Figures and Tables

**Table 1:** Patient characteristics.

Age (Months)	Median (IQR)		67 (31;96.5)
			N (%)
Sex	Total		71 (100%)
	Male		31 (43.7%)
	Female		40 (56.3%)
Side of SNHL	Bilateral		50 (70.4%)
	Unilateral	Total	21 (29.6%)
		Left	11 (15.5%)
		Right	10 (14.1%)
Vestibular hypofunction	Total		41 (57.7%)
	Bilateral		26 (36.6%)
	Unilateral	Total	15 (21.1%)
		Left	4 (5.6%)
		Right	11 (15.5%)

IQR = interquartile range, SNHL = sensorineural hearing loss.

**Table 2:** Proportion of agreement and disagreement between vestibular tests.

Vestibular Tests	VHIT + Caloric Test	VHIT + cVEMP	cVEMP + Caloric Test
Overall agreement (N)	21 (87.5%)	54 (71.1%)	28 (66.7%)
Both tests normal	18 (75.0%)	53 (69.7%)	18 (42.9%)
Both tests abnormal	3 (12.5%)	1 (1.3%)	10 (23.8%)
Overall disagreement (N)	3 (12.5%)	22 (28.9%)	14 (33.3%)
Total ears	24	76	42

VHIT = video head impulse test, cVEMP = cervical vestibular evoked myogenic potential.

**Table 3:** **a**. Overall agreement between VHIT and caloric test per ear. **b**. Overall agreement between VHIT and cVEMP per ear. **c**. Overall agreement between VEMP and caloric test per ear.

**a**				
**Caloric Test**				
VHIT		Normal Test Result	Abnormal Test Result	Total
	Normal test result	18	2	20
	Abnormal test result	1	3	4
	Total	19	5	24
**b**				
**cVEMP**				
VHIT		Normal Test Result	Abnormal Test Result	Total
	Normal test result	53	4	57
	Abnormal test result	18	1	19
	Total	71	5	76
**c**				
**Caloric Test**				
cVEMP		Normal Test Result	Abnormal Test Result	Total
	Normal test result	18	14	32
	Abnormal test result	0	10	10
	Total	18	24	42

VHIT = video head impulse test, cVEMP = cervical vestibular evoked myogenic potential.

**Table 4:** Inter-test agreement between vestibular tests.

Vestibular Tests	Kappa	95% Confidence Interval		Prevalence Index	Bias Index
		Lower	Upper		
VHIT + Caloric test	0.591 *	0.177	1.000	0.625	0.042
VHIT + cVEMP	−0.023	−0.181	0.135	0.684	0.184
cVEMP + Caloric test	0.380 **	0.172	0.588	0.190	0.333

VHIT = video head impulse test, cVEMP = cervical vestibular evoked myogenic potential. * = *p* < 0.05, ** = *p* < 0.005.

## Data Availability

The raw data supporting the conclusions of this article will be made available by the authors on request.

## Abbreviations

The following abbreviations are used in this manuscript:

SNHL	sensorineural hearing loss
VH	vestibular hypofunction
VHIT	video head impulse test
cVEMP	cervical vestibular evoked myogenic potential
